# A Weighted Composite of Endodontic Inflammatory Disease is Linked to a First Myocardial Infarction

**DOI:** 10.3290/j.ohpd.b4586815

**Published:** 2023-11-02

**Authors:** Dan Sebring, Nils-Gunnar Pehrsson, Kåre Buhlin, Peter Jonasson, Henrik Lund, Thomas Kvist

**Affiliations:** a Dentist, Department of Endodontology, Institute of Odontology, Sahlgrenska Academy, University of Gothenburg, Gothenburg, Sweden. Idea, hypothesis, experimental design, data collection, wrote the original draft of the manuscript.; b Consultant, Statistiska konsultgruppen, Gothenburg, Sweden. Experimental design, consulted on and performed statistical evaluation, contributed substantially to discussion, proofread the manuscript.; c Periodontist and Lecturer, Division of Periodontology, Department of Dental Medicine, Karolinska Institute, Huddinge, Sweden. Data collection, contributed substantially to discussion, reviewed and edited the manuscript.; d Endodontist and Lecturer, Endodontics Clinic, Gothenburg, Sweden. Data collection, contributed substantially to discussion, proofread the manuscript.; e Radiologist and Lecturer, Department of Oral Maxillofacial Radiology, Institute of Odontology, Sahlgrenska Academy, University of Gothenburg, Gothenburg, Sweden. Data collection, contributed substantially to discussion, proofread the manuscript.; f Endodontist and Lecturer, Department of Endodontology, Institute of Odontology, Sahlgrenska Academy, University of Gothenburg, Gothenburg, Sweden. Idea, hypothesis, experimental design, data collection, contributed substantially to discussion, reviewed and edited the manuscript.

**Keywords:** cardiovascular disease(s), endodontics, inflammation, periodontal disease(s), risk factors(s), systemic health/disease

## Abstract

**Purpose:**

To explore a weighted composite of endodontic inflammatory disease (EID) as a risk factor for suffering a first myocardial infarction (MI).

**Materials and Methods:**

Seven tooth-specific conditions related to EID were assessed radiographically in 797 patients suffering a first MI and 796 controls. A weighted composite of EID was calculated as the sum of all teeth, excluding third molars. Using maximum likelihood estimation, each condition was assigned a specific weight. With multivariable conditional regression, EID variables, periodontal disease, and missing teeth were assessed as predictors of a first MI.

**Results:**

Periodontal disease (OR 1.38; 95% CI 1.13–1.69, p = 0.0016) and missing teeth (OR 1.03; 95% CI 1.002–1.05, p = 0.034) were related to the risk of a first MI, while none of the EID-related conditions individually were. However, when assessed as an aggregate, a weighted composite of EID (OR 1.97; 95% CI 1.23–3.17, p = 0.0050) and periodontal disease (OR 1.34; 95% CI 1.09–1.63, p = 0.0046) was associated with the risk of MI. Missing teeth did not remain a statistically significant predictor of MI in the final model.

**Conclusions:**

A weighted composite of EID was associated with the risk of MI and strengthens the evidence for a direct connection between oral inflammatory diseases and cardiovascular disorders.

In recent years, inflammation has gained attention as an important contributor to development and progression of cardiovascular disease.^23^ In addition to already well-known risk factors, a systemic low-grade chronic inflammation stimulates atherogenesis, the formation of atheromatous plaques in the blood vessel wall. Inflammation may also increase the risk of plaque rupture, which is the main reason for suffering an acute myocardial infarction (MI).^12^

Periodontal disease (PD),^6^ and more recently also inflammation of endodontic origin,^3^ relates to MI and other manifestations of cardiovascular disease. Additionally, missing teeth, which can be the result of both endodontic inflammatory disease (EID) and PD, is commonly associated with cardiovascular disease.^11,16,22,24,30,31^

Plausible biological mechanisms linking EID and PD to systemic diseases include spread of microorganisms and endotoxins from the site of infection, i.e., the root canal or periodontal pocket. Also, resident cells and recruited immune cells produce pro-inflammatory mediators that may be released into the circulation and contribute to a systemic inflammation.^28^ Carious lesions evoke pulpal inflammation, which may contribute to systemic inflammation.^9^ With improved understanding, prevention and treatment of oral diseases, more teeth are retained at older ages, of which many will have experienced EID and PD and some will, even if treated, present persistent disease. Hence, numerous oral conditions may be assessed as variables related to either EID or PD when studying associations with cardiovascular disease.

Throughout the years, the assessment of PD has been performed by means of classification systems that account for progression and severity, thereby allowing gradation of disease on an individual level.^7^ A similar individual-specific assessment of EID has been lacking. The reason for this is not clear, but possibly the many and varied clinical conditions of EID – i.e., caries, fillings, secondary carious lesions, apical periodontitis, previous root canal treatments and persistent periapical disease – have made a composite measurement difficult to design and explore for validity. In the context of a possible association with systemic disease, summarising an individual’s overall inflammatory burden of endodontic origin would give a more comprehensive description of the condition of EID. Furthermore, it may facilitate a comparison of the importance of these conditions in relation to other inflammatory diseases such as PD.

In a large case-control study, the risk of a first MI was significantly increased in patients with PD even after adjustment for confounding factors.^24^ In further analyses of various potential EID risk factors, untreated caries, periapical lesions, and root fillings, depending on age, were significantly associated with a first MI, but only more missing teeth remained independently associated with such a risk in the complete sample.^25^

The aim of the present study was to explore a weighted composite measure of EID as an independent risk factor for a first MI.

## Materials and Methods

**Fig 1:**
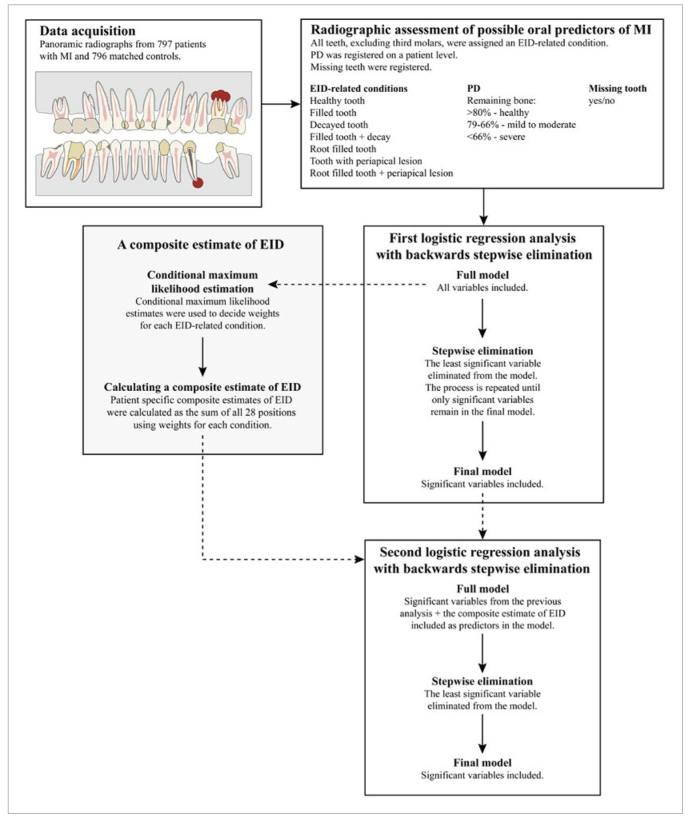
Flowchart of the study. MI = myocardial infarction; EID = endodontic inflammatory disease; PD = periodontal disease.

The layout of the present study is illustrated by the flowchart in Fig 1.

### Data Acquisition and Radiographic Assessment of EID and PD

This study used data from the previously described case-control study PAROKRANK (Periodontitis and its relation to coronary artery disease).^24,25^ In short, 805 individuals with recent experience of a first MI and 805 healthy controls, matched for sex, age and geographical area, were examined for physical and oral parameters, including panoramic radiography.

PD^24^ and EID^25^ were assessed radiographically. Three observers assessed a total of 1593 panoramic radiographs, 797 from MI patients and 796 from controls. The observers were calibrated against a reference standard decided upon by two specialists resulting in an observer-reference agreement of k 0.54, 0.59 and 0.75 when assessing teeth with periapical lesions. All other variables were close to complete agreement.^26^

### Definition of EID and PD

**Fig 2:**
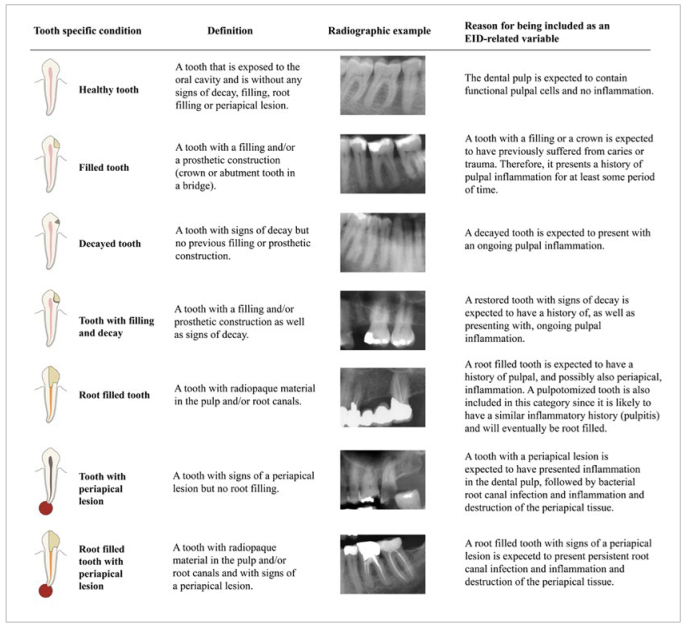
Definitions of the seven conditions relating to endodontic inflammatory disease (EID) that were radiographically assessed. The radiographic examples are sections taken from panoramic radiographs used in the study.

EID was defined as radiographic signs of past or present inflammation in the pulp or the periapical tissue. All teeth, excluding third molars, were assigned the highest of seven hierarchical conditions: 1. healthy tooth; 2. tooth with filling; 3. tooth with decay; 4. tooth with filling and decay; 5. root-filled tooth; 6. tooth with periapical lesion; 7. root-filled tooth with periapical lesion. Definitions, description, and rationale for each condition are presented in Fig 2.

PD was assessed radiographically by measuring from the marginal bone crest to the apex and from the cementoenamel junction to the apex and calculating an arithmetic mean. PD was categorised on an individual level, based on the mean value of all teeth, as healthy (≥80% remaining bone), mild to moderate (79% to 66% remaining bone) and severe (<66% remaining bone).^24^

Missing teeth were assessed as a separate category, because the reason for a missing tooth could relate to either EID, PD or something else.

### Calculating EID as a Composite

The calculated composite measure of EID was intended to be tested as a predictor of MI. As such, the seven EID-related conditions had to be weighted in relation to each other.

Conditional maximum likelihood estimates for each condition were used to assign each of them a specific weight relating to their ability to predict MI. A composite estimate of EID was calculated as the sum of all tooth positions, excluding third molars.

### Statistical Analyses

All variables were first entered into a multivariable conditional logistic regression. Backwards stepwise conditional logistic elimination was applied to reach the best-fit final model, decided by a significance level of 5%. All significance tests were two-sides and conducted at the 5% significance level.

### Ethical Approval

The PAROKRANK study was approved by the Regional Ethics Committee in Stockholm (Dnr:2008/152-31/2). All patients provided written informed consent. PAROKRANK was conducted according to the principles outlines in the Helsinki Declaration. This report conforms to STROBE guidelines.

## Results

### First Backwards Stepwise Elimination: Seven EID-related Conditions, PD and Missing Teeth Included as Predictors for MI

The results of the first logistic regression with backwards stepwise elimination are presented in Table 1. In the full model, only PD (OR 1.36; 95% CI 1.10-1.67, p = 0.0038) and missing teeth (OR 1.03; 95% CI 1.001-1.06, p = 0.040) were related to an increased risk of a first MI. None of the EID-variables were statistically significantly related to the risk of MI.

**Table 1:** Multivariable conditional logistic regression with backwards stepwise elimination of oral predictors and the risk of myocardial infarction

Variables	Full model			Final model		
	OR	95% CI	p-value	OR	95% CI	p-value
Filled	1.01	0.98–1.03	0.71	–	–	–
Decayed	1.10	0.97–1.25	0.12	–	–	–
Filled + decayed	0.95	0.86–1.06	0.34	–	–	–
Root filled	0.99	0.93–1.05	0.73	–	–	–
Periapical lesion	1.07	0.90–1.27	0.45	–	–	–
Root filled + periapical lesion	1.01	0.91–1.14	0.82	–	–	–
PD	1.36	1.10–1.67	0.0038	1.38	1.13–1.69	0.0016
Missing	1.03	1.001–1.06	0.040	1.03	1.002–1.05	0.034

Backwards stepwise elimination identified only PD (OR 1.38; 95% CI 1.13–1.69, p = 0.0016) and missing teeth (OR 1.03; 95% CI 1.002–1.05, p = 0.034) as predictors for MI.

#### Composite estimate of EID: assigning weights to the EID-related conditions

**Fig 3:**
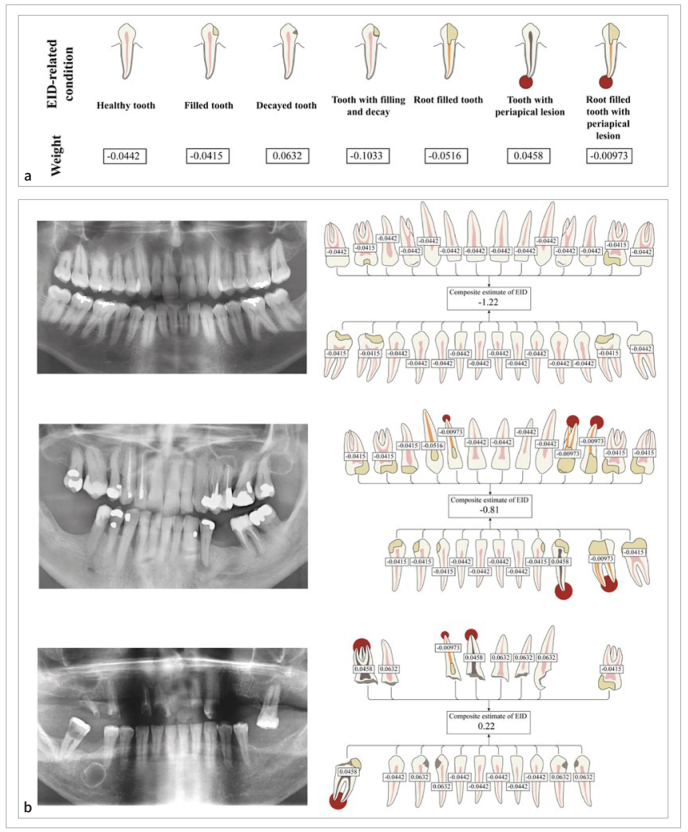
a. The seven conditions relating to endodontic inflammatory disease (EID) and their specific weights, determined by conditional maximum likelihood estimation. b. Three examples of calculating the composite estimate of EID by summarising the specific weights for all EID-related conditions are shown.

Weights, attained from the conditional maximum likelihood estimation, given to each EID-related condition is presented in Fig 3a.

### Second Backwards Stepwise Elimination: PD, Missing Teeth and a Composite Estimate of EID Included as Predictors for MI

The results of the second logistic regression with backwards stepwise elimination and a composite estimate of EID included as a predictor are presented in Table 2. The final model included the composite estimate of EID (OR 1.97; 95% CI 1.23–3.17, p = 0.0050) and PD (OR 1.34; 95% CI 1.09–1.63, p = 0.0046) as statistically significant predictors of MI. Missing teeth did not reach the 5% significance level.

**Table 2:** Multivariable conditional logistic regression with backwards stepwise elimination of PD, the composite estimate of EID and missing teeth and the risk of myocardial infarction

Variables	Full model			Final model		
	OR	95% CI	p-value	OR	95% CI	p-value
PD	1.35	1.10–1.65	0.0042	1.34	1.09–1.63	0.0046
The composite estimate of EID	2.30	0.96–5.49	0.0604	1.97	1.23–3.17	0.0050
Missing teeth	0.99	0.95–1.04	0.6779	–	–	–

#### Composite estimate of EID: patients vs controls

A composite estimate of EID was calculated for all patients and controls by summing up all the specific weight of all teeth, as stated in Fig 3a. Three examples of calculating the composite estimate are illustrated in Fig 3b. Overall, a higher mean (-0.97 vs -1.03) and median (-1.05 vs -1.09) was observed for the patients with a first MI compared to the controls. The maximum value (0.37), indicating greatest EID, was observed in the group of MI patients, while the minimum value (-1.55) was found in the control group.

## Discussion

In a previous publication,^25^ separately assessed EID variables revealed a relationship with the risk of a first MI only in certain age groups. The present study applied conditional logistic regression with backwards stepwise elimination of several EID conditions and confirmed the previous results: only missing teeth and PD remained in the final model that best predicted the risk of MI. However, when summarising several EID-related conditions into a composite, and using conditional maximum likelihood estimation to assign weights to each condition, the resulting composite estimate of EID was statistically significantly related to an increased risk for a first MI. In the final model, the composite estimate of EID as well as PD remained independently associated with MI, while missing teeth lost their role as a risk factor. The findings add support to the hypothesis that EID may be a contributing factor to cardiovascular disease and its sequela, i.e., MI.

EID is hypothesised to play a part in atherogenesis and the development of cardiovascular disease through spread of intracanal infection, endotoxins or pro-inflammatory cytokines.^28^ A reasonable assumption is that the accumulated systemic inflammation is linked to the increased risk of cardiovascular disease in an individual. Therefore, it makes sense to summarise a disease entity in one composite measure rather than to test each manifestation of the disease separately. The seven presently applied EID conditions can be seen as different stages of the same disease. Untreated dental decay results in inflammation of the pulp and subsequent pulp necrosis; apical periodontitis may follow. An early disease manifestation can be treated by removal of caries and filling the cavity, while more advanced disease necessitates endodontic treatment.^5^ Both filled and root-filled teeth may suffer from recurrent or persistent disease.^10,21^ All conditions may in some way be associated with the risk of MI and therefore were included when calculating the composite measure of EID.

Summarising instruments have been used previously for studying the relationship between oral and systemic disease. Mattila et al^17^ assessed dental health using the total dental index (TDI) based on scores given to the number of carious lesions, gingival pocket probing depths, the number of periapical lesions and signs of pericoronitis. Dental health was significantly worse in patients with MI than controls. Decayed, missing and filled teeth/surfaces (DMFT/DMFS) indices, introduced by Klein^15^ as measures of caries experience, have been associated with cardiovascular disease in several studies.^13,22,25,31^

In contrast to previous indices, the present study used conditional maximum likelihood estimation for assigning weights, positive as well as negative, to the conditions when calculating the composite estimate of EID. Healthy teeth and teeth with treated conditions, i.e., filled and root-filled teeth with or without signs of disease, were assigned negative weights while teeth with untreated conditions, i.e., decayed teeth and teeth with untreated apical periodontitis, were assigned positive weights. Accordingly, healthy teeth and treated conditions lowered the individual’s EID estimate and the risk of a first MI, while untreated decay and apical periodontitis increased the risk. However, conclusions about their relative importance must be made very cautiously, since none of the individual variables reached statistical significance.

Intact teeth foster well-being, and are linked to other health-promoting factors, such as good diet,^19^ less smoking^14^ and high socioeconomic status,^29^ which are inversely related to cardiovascular disease. Filled, but otherwise sound, teeth could be argued to no longer suffer from inflammation and therefore not constitute a risk for MI. It must also be acknowledged that teeth can be restored for reasons other than caries and may not necessarily have been affected by pulpal inflammation. Likewise, teeth may be root filled for reasons other than pulpitis and/or apical periodontitis. However, filled teeth with concomitant decay and root-filled teeth with signs of disease also received negative weights. Possibly, filled and root-filled teeth, even if present with signs of disease, indicate a positive attitude to self-care, including regular visits to a dental office, that may be associated with a reduced risk of MI. An alternative explanation could be an overestimation of caries in filled teeth and periapical lesions in root-filled teeth. It may also be hypothesised that fillings and root canal treatment in cases of EID alter the nature of the inflammatory response in the affected tooth and the impact on the cardiovascular system in a beneficial way, even if remnants of disease persist or recur.

In the second logistic regression with backwards stepwise elimination, the composite estimate of EID together with missing teeth and PD was tested as a predictor for MI. The model that best predicted the risk of MI included PD and the composite estimate of EID, while missing teeth did not contribute (Table 2). Missing teeth associated with cardiovascular disease has been a common finding in previous studies.^11,16,22,24,30,31^ Obviously, teeth can be extracted for a variety of reasons, even if inflammatory conditions of endodontic or periodontal origin are the most common.^4,27^ The present findings suggest that it may not be missing teeth per se that increase the risk of MI. Rather, an inflammation, either from EID, PD or both, that is related to a tooth being extracted could be the cause of the observed risk of MI.

Using panoramic radiography for assessment of EID-related variables is not optimal and represents an inevitable limitation of the present study. Although it is considered sufficient in large scale epidemiological studies,^1,18^ panoramic radiography, compared to other methods, suffers from limited sensitivity when diagnosing proximal caries^2^ and apical periodontitis.^8,18^ In the present study, efforts were made to increase the reliability of the radiographic assessments. All observers were calibrated against a reference standard prior to the assessments.^26^ In order to minimise false positive identification which could otherwise impact the ability to detect differences when comparing MI cases and controls, apical periodontitis was defined as a clearly discernible periapical radiolucency. The periapical index (PAI),^20^ originally designed for intra-oral radiography of incisors, was considered unsuitable for panoramic radiography, since it does not allow the detailed assessment necessary for PAI assignment. However, the possible associations between MI and the periapical lesion size and/or a correlation between the radiological features of the lesion and different histologically assessed inflammatory responses (as with PAI) should be addressed in future research.

However, assessing EID as a composite that includes the many stages of pulpal and periapical inflammation also warrants further investigation.

## Conclusion

A weighted composite estimate of EID was independently associated with the risk of a first MI and strengthens the evidence for a direct connection between oral inflammatory diseases (EID and PD) and cardiovascular disorders. From a clinical perspective, the study also supports the premise that prevention and treatment of EID may reduce the risk of cardiovascular disease.
